# A national long-read sequencing study on chromosomal rearrangements uncovers hidden complexities

**DOI:** 10.1101/gr.279510.124

**Published:** 2024-11

**Authors:** Jesper Eisfeldt, Adam Ameur, Felix Lenner, Esmee Ten Berk de Boer, Marlene Ek, Josephine Wincent, Raquel Vaz, Jesper Ottosson, Tord Jonson, Sofie Ivarsson, Sofia Thunström, Alexandra Topa, Simon Stenberg, Anna Rohlin, Anna Sandestig, Margareta Nordling, Pia Palmebäck, Magnus Burstedt, Frida Nordin, Eva-Lena Stattin, Maria Sobol, Panagiotis Baliakas, Marie-Louise Bondeson, Ida Höijer, Kristine Bilgrav Saether, Lovisa Lovmar, Hans Ehrencrona, Malin Melin, Lars Feuk, Anna Lindstrand

**Affiliations:** 1 Department of Molecular Medicine and Surgery, Center for Molecular Medicine, Karolinska Institutet, 171 77 Stockholm, Sweden;; 2 Department of Clinical Genetics and Genomics, Karolinska University Hospital, 171 76 Stockholm, Sweden;; 3 Science for Life Laboratory, Karolinska Institutet Science Park, 171 65 Solna, Sweden;; 4 Department of Immunology, Genetics and Pathology, Uppsala University, 751 85 Uppsala, Sweden;; 5 Science for Life Laboratory, Uppsala University, 752 37 Uppsala, Sweden;; 6 Department of Clinical Genetics and Genomics, Sahlgrenska University Hospital, 413 90 Gothenburg, Sweden;; 7 Division of Clinical Genetics, Department of Laboratory Medicine, Lund University, 221 84 Lund, Sweden;; 8 Department of Clinical Genetics, Pathology and Molecular Diagnostics, Office for Medical Services, Region Skåne, 223 62 Lund, Sweden;; 9 Department of Laboratory Medicine, Institute for Biomedicine, Sahlgrenska Academy, University of Gothenburg, 405 30 Gothenburg, Sweden;; 10 Department of Clinical Genetics, Linköping University Hospital, 581 85 Linköping, Sweden;; 11 Division of Cell and Neurobiology, Department of Biomedical and Clinical Sciences, Linköping University, 581 83 Linköping, Sweden;; 12 Department of Medical Bioscience, Medical and Clinical Genetics, Umeå University, 901 87 Umeå, Sweden;; 13 Department of Pharmacology and Clinical Neurosciences, Umeå University, 901 87 Umeå, Sweden

## Abstract

Clinical genetic laboratories often require a comprehensive analysis of chromosomal rearrangements/structural variants (SVs), from large events like translocations and inversions to supernumerary ring/marker chromosomes and small deletions or duplications. Understanding the complexity of these events and their clinical consequences requires pinpointing breakpoint junctions and resolving the derivative chromosome structure. This task often surpasses the capabilities of short-read sequencing technologies. In contrast, long-read sequencing techniques present a compelling alternative for clinical diagnostics. Here, Genomic Medicine Sweden—Rare Diseases has explored the utility of HiFi Revio long-read genome sequencing (lrGS) for digital karyotyping of SVs nationwide. The 16 samples from 13 families were collected from all Swedish healthcare regions. Prior investigations had identified 16 SVs, ranging from simple to complex rearrangements, including inversions, translocations, and copy number variants. We have established a national pipeline and a shared variant database for variant calling and filtering. Using lrGS, 14 of the 16 known SVs are detected. Of these, 13 are mapped at nucleotide resolution, and one complex rearrangement is only visible by read depth. Two Chromosome 21 rearrangements, one mosaic, remain undetected. Average read lengths are 8.3–18.8 kb with coverage exceeding 20× for all samples. De novo assembly results in a limited number of phased contigs per individual (N50 6–86 Mb), enabling direct characterization of the chromosomal rearrangements. In a national pilot study, we demonstrate the utility of HiFi Revio lrGS for analyzing chromosomal rearrangements. Based on our results, we propose a 5-year plan to expand lrGS use for rare disease diagnostics in Sweden.

Although short-read (sr) genomic analysis approaches such as exome and genome sequencing (GS) have been highly successful in identifying disease-causing genetic variants for diagnostic purposes, the primary focus of analysis remains on single-nucleotide variations (SNVs) and small insertions–deletions (INDELs) (Stranneheim et al. 2021). In contrast, the calling and interpretation of structural chromosomal rearrangements is more challenging (Cameron et al. 2019; Kosugi et al. 2019). Such events, collectively called structural variants (SVs), are defined as genetic variants larger than 50 bp. When SVs involve a repositioning of genetic material either within a chromosome or between chromosomes, they are also referred to as chromosomal rearrangements. These encompass both recurrent and nonrecurrent copy number variations (CNVs, deletions, and duplications) and balanced chromosome abnormalities (translocations, inversions, and insertions). Furthermore, there is growing evidence that complex SVs, which contain multiple breakpoint junctions (BPJs) or consist of more than one simple structural variation in *cis*, are more prevalent than previously assumed (Schuy et al. 2022). A thorough analysis of such events detailing the DNA breakpoints at the nucleotide level is crucial for comprehending the disease-causing and rearrangement-generating mechanisms. This level of understanding is necessary for effective personalized clinical management and genetic counseling (Tesi et al. 2023).

While srGS is well established as a first-line diagnostic test, with the potential to capture pathogenic SVs previously identified by traditional methods (Lindstrand et al. 2019), it still has limitations. The existing srGS SV pipelines lack the ability to produce high-quality genome assemblies necessary to resolve complex disease-causing SVs. In this context, long-read genome sequencing (lrGS) has emerged as a promising alternative with the potential to capture the complete scope of structural genomic variation in the human genome (Audano et al. 2019; Eisfeldt et al. 2019, 2021). Indeed, lrGS provides a comprehensive approach for characterizing various types of SVs identified in a clinical genetic laboratory, whether discovered through traditional methods or srGS. With the introduction of lrGS using highly accurate consensus reads (HiFi reads), it has been demonstrated that SNV and INDEL calls have precision and recall rates that match or exceed what is obtained from srGS (Wenger et al. 2019; Mahmoud et al. 2024). In addition, HiFi reads dramatically increase the ability to detect SVs (Mahmoud et al. 2024). Due to the several kb long sequence reads, lrGS offers unique possibilities to study regions in the human genome that are not easily captured by other genomics technologies, such as highly repetitive or homologous regions. Furthermore, sequencing of the native DNA also provides methylation information. With decreasing cost, it is increasingly attractive to use lrGS in unsolved rare disease cases. Yet, lrGS differs markedly from short-read sequencing and various factors should be considered before its clinical implementation. These include the quality and quantity of DNA starting material, intricate specifics related to sequencing including read quality, total coverage, and read length, as well as the complexities tied to data analysis, visualization, and the eventual clinical interpretation.

Recognizing the promise and challenges of lrGS, we embarked on a national pilot study using the PacBio Revio system. We leveraged Genomic Medicine Sweden (GMS), a national initiative designed to implement precision medicine throughout the regionally organized healthcare system (Fioretos et al. 2022) with the aim to validate lrGS for clinical digital karyotyping of unresolved SVs nationwide. The included samples originated from all university hospital regions in Sweden and the DNA was not necessarily collected using lrGS-optimized protocols. This approach mirrored the typical sample quality and preparation found in most of Sweden's hospitals and biobanks today, thereby giving us a realistic view of the potential and challenges of lrGS in clinical diagnostics.

## Results

### High-quality long-read genome data are obtained from clinical DNA samples

**Figure 1.**
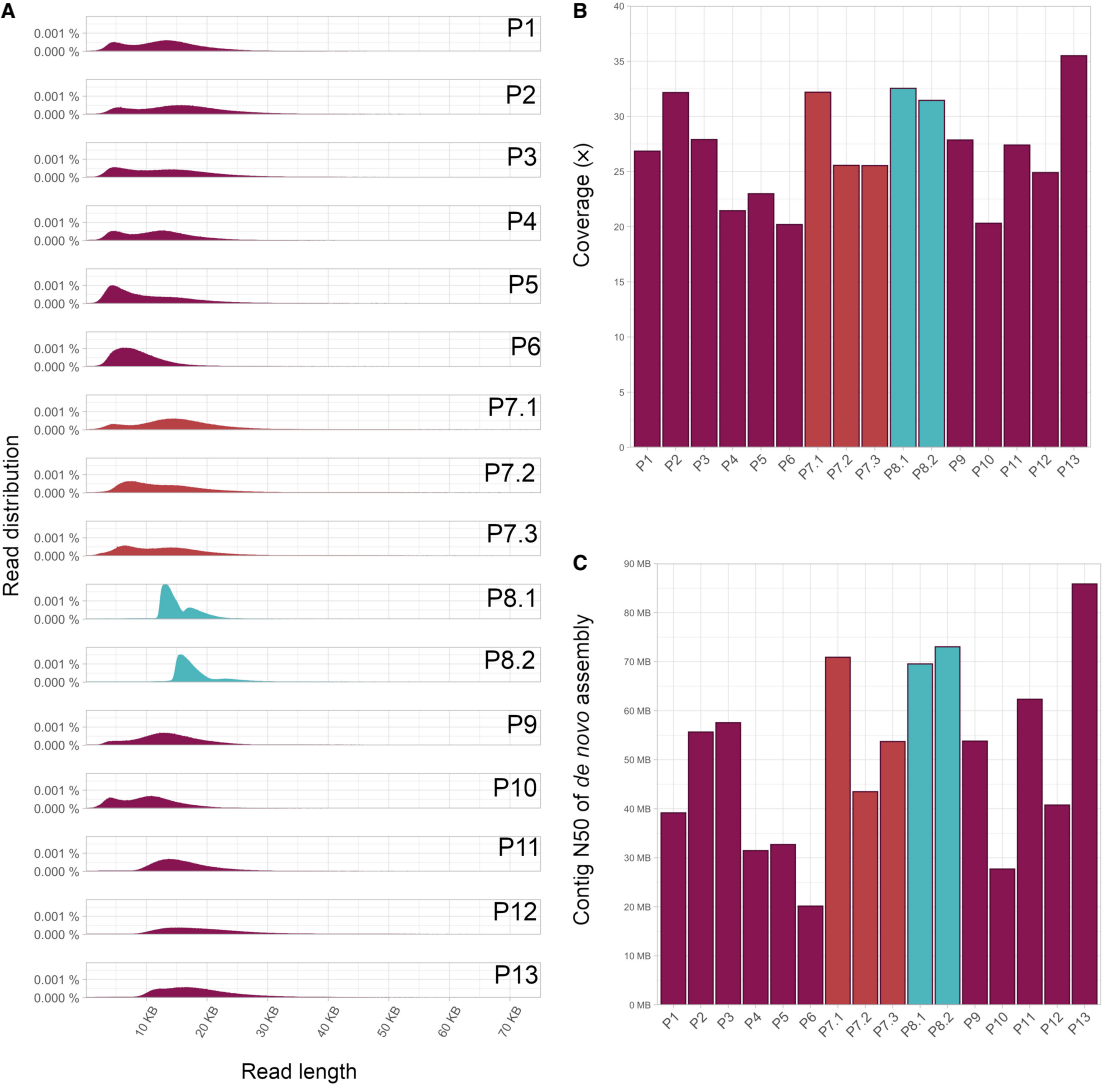
Quality measures of lrGS. (*A*) Read length distribution, (*B*) coverage, and (*C*) N50 of de novo assembly.

All individuals were sequenced on one Revio 25M ZMW SMRT Cell, generating at least 20× coverage of high-quality (HiFi) reads for all samples (range 19.9–35.5×), with mean read length ranging from 8.3 to 18.8 kb (Fig. 1A,B; Supplemental Table S1). Two samples (P8.1 and P8.2) were obtained from high-molecular-weight DNA extraction of fresh blood, enabling a more efficient size selection during library preparation. Gel-based size selection was performed not only on those two samples but also on three regular DNA samples (P11, P12, and P13). Contrary to expectations, all five samples achieved similar levels of high coverage and longer read lengths, with P13 reaching the highest overall throughput of >35× coverage and the best assembly results. This indicates that the regular DNA extractions are suitable for HiFi sequencing and can be used to generate the best possible data from a single SMRT Cell. Variant calling resulted in an average of 4.9 million SNVs and small INDELs per individual (Supplemental Tables S2, S3). Of the 4.7 M SNVs with a variant quality above 20, 96% are present in gnomAD (Supplemental Table S4; Chen et al. 2024). We further performed de novo assembly for all individuals and the contig N50 values are shown in Figure 1C.

### Identification of chromosomal rearrangements

The lrGS analysis could identify 14 of the 16 unique chromosomal rearrangements that were present in the 16 individuals sequenced. Of the two rearrangements that fully eluded detection, one was mosaic (P10) and the second affected the acrocentric p-arm of Chromosome 21, a known repetitive genomic region (P13) (Table 1). The mosaic tri- and tetrasomy on Chromosome 15 in individual P2 was characterized as a copy number gain (between 2× and 5×) in the long-read CNV-calls, although a clear distinction between the tri- and tetrasomy could not be made. While the starting point could not be distinguished from the centromere, the location of the end point was approximated between Chr15:32176000–32346000, with the exact breakpoint located within a segmental duplication (Supplemental Fig. S1). The remaining samples were then subdivided depending on whether they carried an interchromosomal or intrachromosomal rearrangement.

**Table 1.** Rearrangement details and clinical features of patients included in the present study

Case ID	SV structure	ISCN 2020/HGVS nomenclature	Main phenotype	Clinical assessment
P1	Unbalanced translocation, DEL	seq[GRCh38] der(4)t(4;9)(q34.3;p23), del(16)(p12.1p11.2) NC_000004.12:g.181414917_qterdelins[NC_000009.12:g.pter_13043905] NC_000016.10:g.28455071_29508000del	Mild ID and obesity. Strabismus (exotropia), hypermetropia, and astigmatism. Features include a large forehead, narrow eye slits, prominent labial furrows, and skin hyperpigmentation.	Pathogenic/pathogenic
P2	Isodicentric chromosome	seq[GRCh38] 15q11.1q13.2(19799420_30095350x3-4)dn,15q13.2q13.3(30521460_32201830x2-3)dn	Suspected global developmental delay. Epilepsy, spasms.	Pathogenic
P3	DEL-INV-DEL	seq[GRCh38] 22q13.33q13.33(50624868_50626276)x0 NC_000022.11:g.50618055_50626277delins[NC_000022.11:g.50624361_50624868inv] NM_000487.6(ARSA):c.[857_*277del];[857_*277del] p.(Pro286_Ala509delins8)	Normal development in the first year. Progressive loss of functions, muscle weakness, areflexia, ptosis, and swallowing difficulties in the second year.	Pathogenic
P4	DUP-TRIP-QUAD-TRIP-DUP-NML-DEL, DEL-INV-DEL	seq[GRCh38] der(2)ins(2)(p25.1p25.2p25.2)ins(2)(p25.1p25.1p25.2)ins(2)(p25.1p25.2p25.2)del(p25.1p25.1), del(q21.2)ins(2)(q21.2q22.1q21.2) NC_000002.12:g.7246507_7734100delins[g.5620671_6248808inv;g.5549092_7241289inv;g.5858296_6246303],g.132954122_141163928delins[g.133434126_137703180inv]	Syndromic craniofacial condition	Likely pathogenic/likely pathogenic
P5	DEL-INV-NML-DUP-NML-DUP	seq[GRCh38] der(3)del(3)(p14.3p14.2)inv(3)(p14.2p14.2)ins(3)(p14.2q22.2q22.2)ins(3)(p12.2q24q24) NC_000003.12:g.54518907_80981660delins[g.59407900_63614153inv;g.135767535_135891958;g.63614153_80981660inv]	Motor delay	Pathogenic
P6	Translocated insertion	seq[GRCh38] der(X)del(X)(q28q28)ins(X;9)(q28;q22.33q22.33) NC_000023.11:g.153724706_153779639delins[NC_000009.12:g.97596764_97598236inv]	Adrenoleukodystrophy	Pathogenic
P7.1	Translocation, INV	seq[GRCh38] t(1;10)(p36.2;q24), inv(2)(q32.2q33.2) NC_000001.11:g.19783172_qterdelins[NC_000010.11:g.pter_95395335] NC_000010.11:g.pter_95395328delins[NC_000001.11:g. 19783105_qter] NC_000002.12:g.189694535_202576083inv	Recurrent miscarriages	Pathogenic Pathogenic
P7.2	NA	NA	Healthy father of P7.1	NA
P7.3	NA	Same as P7.1	Healthy mother of P7.1	NA
P8.1	DUP-INV-DUP	seq[GRCh38] der(X)ins(X)(p22.2q28q28)inv(X)(p22.2q28)ins(X)(q28p22.2p22.31) NC_000023.11:g.9768910_154113036delins[g.9420014_154208530inv]	Short stature	Pathogenic
P8.2	NA	Same as P8.1	Healthy, normal height	NA
P9	Translocation	47,XX,t(X;9)(p22;q12)[27]/46,X,t(X;9)(p22;q12)[3].seq[GRCh38] t(X;9)(p22.33;q21.13) NC_000009.12:g.pter_(75862011)delins[NC_000023.11:g.3044233_qter] NC_000023.11:g.3044228_qterdelins[NC_000009.12:g.pter_(75862011)]	Thrombocytopenia, early menopause, learning difficulties	Likely pathogenic
P10	Ring chromosome, DEL	46,XY,r(21)(p11q22)[9]/46,XY, del(21)(q22.3)[4]/46,XY[12]	Infertility, oligospermia	VUS
P11	DUP-NML-DEL	seq[GRCh38] der(X)ins(X)(q22p11.21p22.33)del(X)(q22q28) NC_000023.11:g.101431832_qterdelins[g.pter_55349282inv]	ID, delayed puberty	Likely pathogenic
P12	Complex translocation	seq[GRCh38] t(1;4;6;4)(p32.2;q21.1;p22.3;q22.3q24), del(6)(p12.3p12.3) NC_000001.11:g.pter_58205785delins[NC_000004.12:g.106505092_qterinv] NC_000004.12:g. 80390384_qterdelins[NC_000006.12:g.pter_20052376inv] NC_000006.12:g.20052374_pterdelins[NC_000004.12:g.94218568_106505089inv;NC_000001.11:g.20052374_pter] NC_000006.12:g. 48002694_49160076 del	Neonatal hypotonia, ID, short stature	Pathogenic
P13	Unknown insertion	46,XY,add(21)(p1?3)	Infertility, oligoasthenozoospermia	VUS

(SV) structural variant, (DEL) deletion, (INV) inversion, (DUP) duplication, (TRIP) triplication, (QUAD) quadruplication, (NML) normal (copy number), (NA) not applicable, (ISCN) the international system of for human cytogenomic nomenclature, (HGVS) human genome variation society nomenclature, (DEL) deletion, (INV) inversion, (DUP) duplication, (TRIP) triplication, (QUAD) quadruplication, (NML) normal copy number, (ID) intellectual disability, (VUS) variant of uncertain significance. Unresolved rearrangements are shown in gray.

#### Translocations and inversions

**Figure 2.**
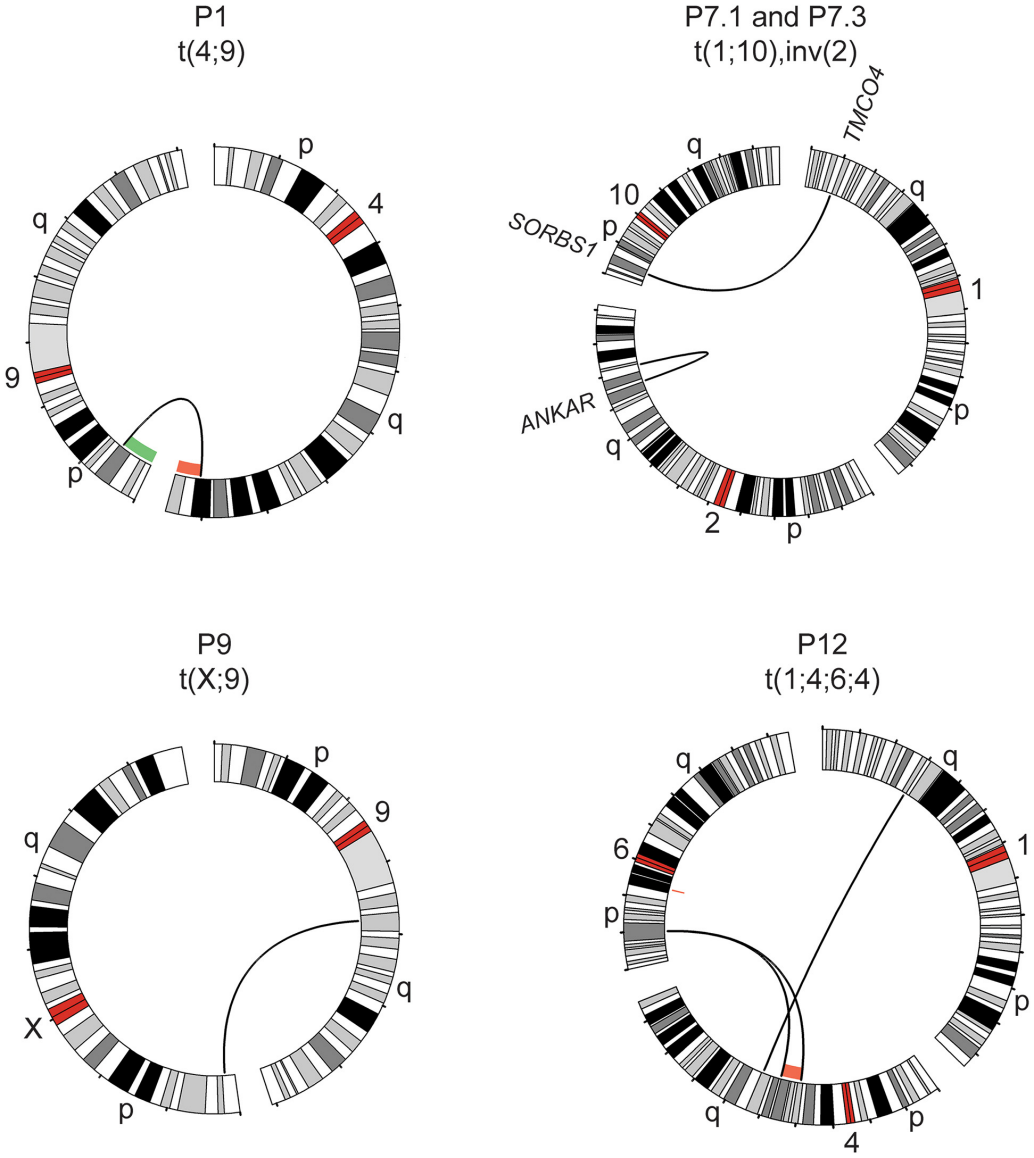
Translocations and inversions identified with lrGS. Circos plots of rearrangements detected in four cases using lrGS: a t(4;9) in P1, a t(1;10) and inv(2) (P7.1 and P7.3), a t(X;9) (P9), and a t(1;4;6;4) (P12). A green/red line indicates copy number gain/loss, respectively. Genes disrupted are indicated at the breakpoint.

Three translocations and one inversion were characterized in four individuals (Fig. 2; Table 1) with a mother and daughter both carrying the same two balanced events (P7.1 and P7.3). All variants were fully resolved, but the translocation between Chromosome X and Chromosome 9 (P9) was only detectable using the Telomere-to-Telomere (T2T) assembly for analysis. A secondary analysis in hg38 revealed a call of a t(X;5) in that sample, indicating that the breakpoint 9 region is missing in GRCh38. In individual P1 the three SVs (two deletions and one duplication) detected before lrGS were in fact two separate rearrangements; one recurrent 16p deletion and one unbalanced translocation between Chromosome 4 and Chromosome 9 (Fig. 2). Finally, we were able to delineate a complex chromosome translocation involving Chromosomes 1, 4, and 6 (P12). The analysis detected four BPJs, a 13.8 Mb deletion on Chromosome 4 as well as an independent 1.2 Mb deletion on Chromosome 6 located centromeric of the Chromosome 6 breakpoint (Fig. 2; Table 1).

#### Complex intrachromosomal rearrangements

**Figure 3.**
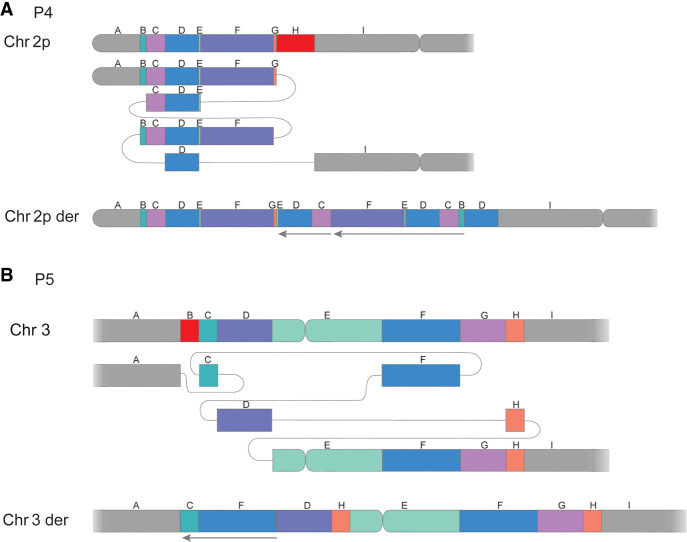
Subway plots of two complex rearrangements: (*A*) a complex dup-trip-quad-trip-dup-del on Chromosome 2 observed in P4 and (*B*) a clustered CNV on Chromosome 3 detected in P5. Deleted segments are shown in red, and arrows mark inverted segments.

In addition to the complex translocation discussed above, six intrachromosomal complex rearrangements were detected and resolved in five unrelated individuals, all of which were unbalanced (Table 1). Notably, the same inv(X) was detailed in a mother and daughter pair (P8.1 and P8.2). The highest complexity level was identified in P4 where two complex events were identified, one DUP-TRIP-QUAD-TRIP-DUP-DEL and one DEL-INV-DEL (Fig. 3A; Table 1). The complexity found in P4 was tightly followed by P5, who carries a complex rearrangement consisting of two duplications, one deletion, as well as an inverted copy number neutral segment (Fig. 3B; Table 1). In contrast, the simplest rearrangements consisted of DEL-INV-DEL (P3 and P4) or DEL-INS-DEL (P6) (Supplemental Fig. S2). Finally, we resolved a terminal invDUP-NML-DEL on Chromosome X (P11), mediated by matching *Alu*Y elements and harboring a 291 long stretch of microhomology (Table 2).

**Table 2.** Junction characteristics of identified rearrangements, one case was only detected in T2T

Sample ID	BPJs	ChrA	posA	ChrB	posB	Strand	Gene posA	Gene posB	Breakpoint insertions (nt)	Repeat A	Repeat B	Microhomology (nt)
**GRCh38 (hg38)**												
P1	1	4	181414917	9	13043905	+/−	–	–	–	L1	L1	–
	1	16	28455071	16	29476612	+/+	–	–	–	*Alu*J**o	SVA_D	–
P3	2	22	50618055	22	50624868	+/−	–	***ARSA***	2	tigger1	MIR	–
		22	50624361	22	50626277	+/−	***ARSA***	***ARSA***	–	*Alu*sp	–	2
P4	4	2	5549092	2	5858296	+/−	*XR_001739261.1*	–	–	–	L1PA7	4
		2	5620671	2	7241289	+/+	–	–	9	–	–	–
		2	6246303	2	7734100	+/+	–	–	–	L1M5	–	2
		2	6248808	2	7246507	+/−	–	–	–	L1ME4b	–	3
	2	2	132954122	2	137703180	+/−	*NCKAP5*	–	–	MLT1K	L1PA16	1
		2	133434126	2	141163928	+/−	*NCKAP5*	*LRP1B*		–	–	11
P5	5	3	54518907	3	63614153	+/−	*CACNA2D3*	*SYNPR*	–	–	MamRep38	–
		3	59407900	3	135767535	+/−	*XR_002959675.1*	–	–	–	MER101	1
		3	63614153	3	145600155	+/−	*SYNPR*	–	–	MamRep38	L2A	–
		3	80981660	3	135891958	+/−	–	–	–	MSTA-int	L2a	1
		3	80981660	3	148110490	+/+	–	–	1	MSTA-int	MER58A	–
P6	2	9	97596764	X	153779639	+/−	*TMOD1*	–	–	–	–	1
		9	97598236	X	153724706	+/−	*TMOD1*	*BCAP31*	12	*Alu*SX1	–	–
P7.1 P7.3	4	1	19783172	10	95395335	+/−	*TMCO4*	*SORBS1*	7	MIRb	–	–
		1	19783105	10	95395328	+/−	*TMCO4*	*SORBS1*	5	MIRb	–	–
		2	189694535	2	202576085	+/−	–	*ANKAR*	1	–	L1PA17	–
		2	189694533	2	202576083	+/−	–	*ANKAR*	–	–	L1PA17	1
P8.1 P8.2	2	X	9420014	X	154113037	+/−	XR_001755796.1	–	28	*Alu*Jr	*Alu*Sx1	–
		X	9768909	X	154208530	+/−	*GPR143*	–	31	*Alu*Sz6	*Alu*J**o	–
P11	1	X	55349282	X	101431831	+/−	–	*ARMCX4*	–	*AluY*	*Alu*Y	290
P12	5	4	80390384	6	20052376	+/−	*CFAP299*	–	–	L1MA9	*Alu*Sq2	1
		4	94218568	6	20052374	+/−	*SMARCAD1*	–	–	*AluSz*	*Alu*Sq2	1
		1	58205787	4	106505089	+/−	*DAB1*	–	–	–	THE1D	–
		1	58205785	4	106505092	+/−	*DAB1*	–	–	–	THE1D	2
		6	48002694	6	49160076	+/+	*PTCHD4*	–	–	L1PA4	–	3
**T2T**												
P9	2	9	75862011^a^	X	3044233	+/−	–	–	–	(AATGG)n	ERVL-MaLR	2
		9	75862011^a^	X	3044228	+/−	–	–	990	(TTCCA)n	ERVL-MaLR	–

(BPJ) breakpoint junction, (Chr) Chromosome, (pos) position, (nt) nucleotide; bold text indicates a disease-causing gene.

^a^Estimated location.

### Breakpoint junction analysis

In 13 of the rearrangements, the BPJs were characterized at nucleotide resolution, allowing for a detailed analysis of insertions, repeat elements and microhomology, and disrupted genes (Table 2; Supplemental Table S5). Comparing the Sniffles SV calls to the calls from our de novo assembly pipeline, we find that the de novo workflow detects 30 of the 31 BPJs detected by Sniffles (Table 2; Supplemental Table S5). Notably, we find a great diversity of such genetic signatures in the analyzed cases. In three rearrangements, the breakpoints contain matched repeat elements: L1 in the t(4;9) (P1) and various *Alu* elements in the inv(X) (P8.1 and P8.2) as well as in P11. In individual P4 (Table 2) microhomology of 1–11 nt was observed in five of the six BPJs and the final BPJ contains a 9 nt insertion. Insertions larger than 2 nt were detected in three additional rearrangements (Table 2); a complex translocational insertion (P6) (Supplemental Fig. S2), a t(1;10) (P7.1 and P7.3) (Fig. 2) and a t(X;9) (P9) (Fig. 2). The final two rearrangements, a DEL-INV-DEL on Chromosome 22 (P3) (Supplemental Fig. S2) and a complex DEL-INV-NML-DUP-NML-DUP on Chromosome 3 (P5) (Fig. 3B), contained neither microhomologies, matched repeats, nor larger insertions (Table 2).

### Characterization of background structural variants

**Figure 4.**
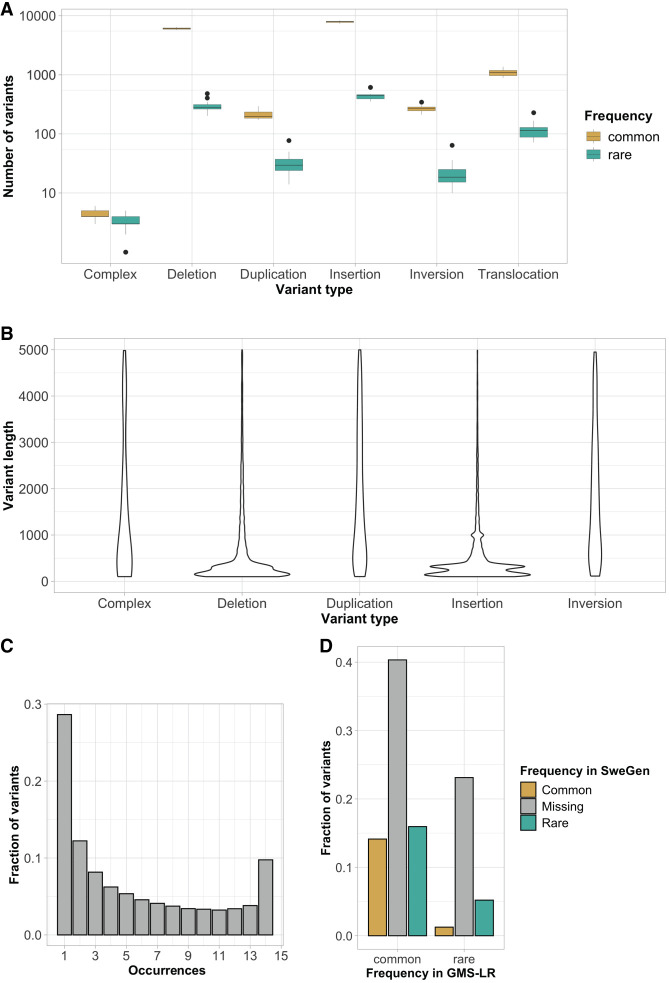
Characterization of background SVs. (*A*) Boxplot illustrating the number of SVs per type (common in yellow and rare in green). (*B*) Violin plot of SV length per SV type (excluding SV > 5 kbp). (*C*) Allele frequency histogram. (*D*) Comparison of allele frequencies between the GMS long-read cohort and SweGen srGS SV database (common in yellow, missing in gray, and rare in green).

Next, we assessed the SV burden in the 14 unrelated samples of the GMS Rare Disease (GMS-RD) lrGS cohort. On average, we detected 16 905 SVs larger than 100 bp per individual that were subdivided into complex (0.05%), deletions (41%), duplications (1%), insertions (51%), inversions (1%), and break end (translocations, mobile elements, transpositions, and reference misalignments) (5%) (Fig. 4A; Supplemental Table S6). In general, the detected SVs were small, with 75% of SV below 1000 bp and a peak at 300 bp representing the *Alu* insertions and deletions (Fig. 4B; Supplemental Table S6). Most variants are present in more than one of the 14 unrelated individuals and only 9% are unique (Fig. 4C; Supplemental Table S6). Furthermore, very few unique SVs affect exons of 1467 genes in the panel app intellectual disability gene panel v6.0 (average 8 per individual, range 1–14). Finally, when comparing to the SweGen SV database (Ameur et al. 2017) containing background SVs in 1000 Swedish individuals assessed from srGS data, ∼70% of the lrGS SV calls are novel and not present in the SweGen data set (Fig. 4D).

## Discussion

In this national collaborative project, we not only demonstrated the usefulness of lrGS as a clinical follow-up analysis of SVs detected by other methods, but we have also taken the first steps toward clinical long-read diagnostics of rare diseases in our nation. By utilizing the expertise in our different regions, we have developed a comprehensive workflow covering sample collection, DNA preparation as well as sequencing, and data analysis.

We fully characterized 13 of the 16 unique chromosomal rearrangements in the GMS-RD cohort. For all cases, both an SV and a CNV (read depth) analysis were done. Specifically, lrGS missed two Chromosome 21 rearrangements (P10 and P13) and one case of mosaic partial tri- and tetrasomy on Chromosome 15 (P2), was only revealed through read depth analysis. This underscores the necessity of including read depth callers for a comprehensive lrGS analysis. The P2 rearrangement was first detected in a clinical diagnostic laboratory by srGS, using read depth analysis, and no cytogenetic analysis was performed. However, the read depth pattern suggested an isodicentric Chromosome 15. The coverage in the two undetected events (20× and 35×, respectively, for P10 and P13) might have affected our sensitivity. In P10, the 21q deletion is estimated to be present in <20% of the cells from the array analysis and in theory should have been supported by two reads and requiring a total of three flow cells to obtain >5 reads. However, the mappability of the data may also influence the result, and other analytical approaches such as a pangenome analysis or optical genome mapping and hybrid assembly may be required to resolve the derivative chromosome structure (Eisfeldt et al. 2021; Schuy et al. 2024). This might be especially relevant in P13 (where the coverage was good) since the added genetic material is located in a repetitive region longer than the read length.

By resolving the structure of the BPJs in 13 rearrangements (Table 2), we uncovered additional complexities compared to the original analysis important for a complete clinical interpretation. In addition, we identified diverse mechanisms of SV formation. Matched *Alu* repeats were present at the BPJs in two rearrangements (P8 and P11), a pattern indicative of *Alu*/*Alu*-mediated rearrangements (Nazaryan-Petersen et al. 2016, 2018; Song et al. 2018; Pettersson et al. 2020). The Chromosome 16 deletion observed in P1 has both BPJs in segmental duplications as expected in a recurrent nonallelic homologous recombination (NAHR) mediated event (Carvalho and Lupski 2016). In five complex SVs from four individuals (P3, P4, P6, and P7) both microhomology and insertions were observed at the breakpoints (Table 2), features indicative of replicative mechanisms such as Fork Stalling and Template Switching (FoSTeS)/microhomology-mediated break-induced replication (MMBIR) (Hastings et al. 2009; Carvalho and Lupski 2016). However, the largest insertion, 990 nt present at the BPJ of the t(X;9) in P9, consists mainly of TTCCA repeats that may be due to background variation in this repeat, and not caused through the formation of the translocation.

**Figure 5.**
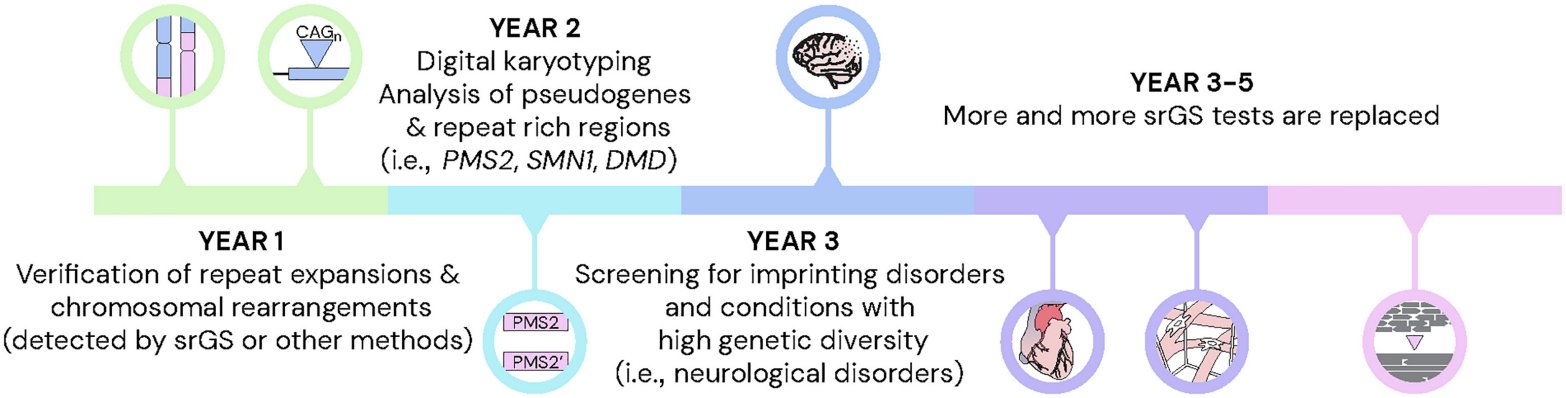
Toward lrGS as a first tier diagnostic test in rare disease. A 5-year time line with the expected development of long-read sequencing in the clinical setting.

In a broader context, the current case series showcases how SVs represent a promising start for the introduction of lrGS into clinical diagnostics. However, a more complete transition to lrGS will take some time. As such, the GMS Rare Disease (GMS-RD) group has drafted a 5-year roadmap, during which new lrGS diagnostic tests successively will be implemented in Sweden (Fig. 5). To accomplish this, there will be a need to streamline the workflows for DNA extraction, library preparation, sequencing, bioinformatic analysis, and variant interpretation. Based on this pilot study, we here highlight some of the main challenges and potential solutions for the wider implementation of lrGS into clinical routine.

Starting with DNA extraction, our results show that lrGS with PacBio Revio performs well with blood samples extracted in routine diagnostics. We obtained >20× coverage for all the samples, which is likely enough for calling of both small and large variants (Harvey et al. 2023; Kucuk et al. 2023). The two samples where HMW DNA was extracted from fresh blood (P8.1 and P8.2) gave slightly more data on average, but we also obtained high yields from the three routine DNA samples (P11, P12, and P13) where we had enough DNA to perform gel-based size selection (Supplemental Table S3). For routine clinical implementation, we, therefore, propose to use the regular DNA extraction protocols that are already implemented at the hospitals, which will reduce the turn-around time and cost.

The second step is library preparation, which was performed manually. Although feasible for a small number of samples, it will not scale in clinical routine, where thousands of rare disease patients and relatives are expected to be tested yearly in Sweden (Fioretos et al. 2022). We, therefore, face the need to implement automated library preparation on liquid handling systems. This will be challenging for certain library preparation steps, such as the shearing and size selection that are not part of a typical srGS library preparation.

Regarding the long-read sequencing, the main limitations are capacity and costs. The PacBio Revio system used in this study has a theoretical capacity of ∼1300 human genomes per year which is ∼15 times less than the NovaSeq X platform. Nevertheless, advancements in technology may lower costs and increase throughput, allowing for more patient testing. Introducing barcoding could also enable sequencing multiple samples per run, improving efficiency. Particularly, for certain rare disease patient groups, such as those with neurological disorders caused by repeat expansions, targeted gene panels might be a more cost-effective approach (Höijer et al. 2018; Stevanovski et al. 2022).

Long-read sequencing generates large amounts of data that need to be processed with streamlined bioinformatics pipelines. For clinical implementation, we need robust and efficient pipelines. Moreover, since bioinformatics is a rapidly evolving research area, it is crucial that the pipelines can be modified as new software emerges. We see great value in processing samples from different hospitals with a similar analysis pipeline, since this will enable joint downstream analyses and comparisons between larger patient groups. For this purpose, we have established a common pipeline (https://github.com/genomic-medicine-sweden/nallo) for the lrGS analysis of rare disease patients in Sweden. All regions in the country have access to the pipeline and can contribute to its continuous development.

Finally, the called variants undergo prioritization and interpretation. In this pilot, the focus was on SVs, and for all cases, there was prior information about the chromosomal rearrangement. In the future, when lrGS is applied as a first-line test for rare disease diagnostics the data analysis will be more challenging. In particular, there is a need to establish a national lrGS reference data set for effective filtering of nonpathogenic variants. However, even with a database containing 14 unrelated long-read genomes, we can reduce the number of unique SVs by 91% (Supplemental Table S4), but for clinical implementation, larger databases will be needed. A few lrGS databases, including the 1000 Genomes Project ONT Sequencing Consortium (1KGP-ONT) (Gustafson et al. 2024) and the Consortium of Long Read Sequencing Database (ColoRSdb, HiFi data) (https://doi.org/10.5281/ZENODO.11511513), are available; however, an in-house database is likely necessary for optimal filtering. To this end, we are planning to sequence a cross section of the Swedish population. The initial reference data set will consist of at least 100 individuals capturing most of the common variants in the population. Ideally, ethnic minority groups should also be included although it might be possible to obtain such data through international collaborations. In addition to a national reference data set, other lrGS resources such as HPRC (Liao et al. 2023) and 1KGP (Gustafson et al. 2024) will be valuable. It may also be necessary to utilize multiple reference genomes, or even a human pangenome graph (Liao et al. 2023), since we noticed here that T2T-CHM13 (Nurk et al. 2022) enables the detection of SV breakpoints not seen in GRCh38.

Variant calling is followed by variant prioritization, enabling the identification of the pathogenic variant(s). During this process, visualization is an important tool. The Integrative Genomics Viewer (IGV) (Robinson et al. 2011), often used for short reads, is mainly designed for the visualization of small variants (INDELs and SNVs), and for SVs identified by lrGS, alternative methods will be required. In this study, we generated subway and Circos (Krzywinski et al. 2009) plots for this purpose, but these might not be suitable for all types of variants. Particularly, specialized tools should be implemented to visualize tandem repeats (De Coster et al. 2024; Dolzhenko et al. 2024).

When a new candidate variant has been detected and visually inspected, its role in the disease needs to be established. We expect that many of the novel variants detected through lrGS will be of unknown significance, since they have not been observed with previous genome technologies, and therefore it will be important to obtain good annotations and functional predictions. To some extent, the same tools as for srGS can be used for functional prediction, but it may be a challenge to understand the potential consequences of complex SVs, especially those located within noncoding regions. For that reason, it may be necessary to focus on the most obvious results and leave some of the uncertain diagnoses for later. As the databases and annotations grow, we can then revisit those patients and hopefully give a correct diagnosis.

Altogether, for the 14 detected rearrangements, the lrGS analysis has not yet led to a clinical reclassification (10 pathogenic, 4 likely pathogenic) (Table 1). In two cases, the SVs caused monogenic diseases; the homozygous 22q13.33 deletion in P3 results in the loss of *ARSA* (Metachromatic Leukodystrophy, MIM 250100), and the hemizygous Xq28 deletion in the loss of *ABCD1* in P6 (Adrenoleukodystrophy, MIM 300100); however, this was known before lrGS. For P10 and P13, whose Chromosome 21 rearrangements remain unresolved, it is still unclear if the clinical symptoms (Infertility and oligospermia/oligoasthenozoospermia) are a result of the cytogenetic rearrangements.

Another potential benefit of lrGS is the ability to call methylation. However, currently, it is challenging to go beyond targeted analysis of regions of interest. The horizon to replace methylation-sensitive MLPA analysis, used in imprinting disorders such as Prader–Willi syndrome (MIM 176270) or to assess skewed X-inactivation, is likely short (Johansson et al. 2023; Yamada et al. 2023; Geysens et al. 2024). However, for a more comprehensive analysis, a large control data set is required. The rare disease community will need to collaborate and share data from methylation analyses conducted with lrGS to establish informative methylation sites, as well as analysis protocols and standard operating procedures. Ultimately, the diagnostic value of methylation analysis needs to be assessed across different rare disease cohorts.

In summary, we demonstrate that by coordinating our local efforts and working together in the GMS Rare Diseases consortium, we were able to build the tools and workflows necessary to validate lrGS for digital karyotyping in the entire nation. Even though there is still quite some work to be done for the full clinical utility of lrGS, our preliminary results show that there is no question that lrGS will provide benefits for rare disease diagnostics. With the plan we have laid out, we hope to have the methods set up and running at scale for rare diseases within a 5-year period.

## Methods

### Study subjects

All Swedish university regions are affiliated with the GMS working group for rare diseases (GMS-RD). In a nationwide lrGS pilot study samples from individuals with seemingly complex SVs were recruited from all healthcare regions. The multicenter study was approved by the Swedish Ethical Review Authority (2019-04746) and written informed consent was obtained from each participating individual or their respective legal guardians. Altogether, 16 samples were sequenced including 11 proband singletons, one duo, and one trio. The duo was a mother and daughter from a previously published family with several carriers of the balanced and unbalanced versions of a complex inversion (Pettersson et al. 2020). The two samples (P8.1 and P8.2) both had the balanced inversion but presented with variable severity (short stature vs. normal height). The trio included a proband with multiple miscarriages that was a carrier of an inversion and a translocation. Her healthy parents, not previously analyzed, were included. For the duo and trio high-molecular preparation had been performed before lrGS, using the Nanobind CBB kit (Pacific Biosciences) while following protocol Nanobind HMW DNA extraction—mammalian whole blood (PN 102-573-500, REV01) and the SP blood and cell culture DNA isolation kit (Bionano Genomics) following SP Blood and Cell Culture DNA Isolation Protocol v2 (document no. 30398, revision B), respectively. The remaining DNA samples were retrieved from clinical biobanks and obtained using standard extraction protocols (Supplemental Table S7). The clinical relevance of the SVs was classified according to the American College of Medical Genetics and Genomics (ACMG) guidelines (Riggs et al. 2020). Details on the included individuals are given in Table 1.

### Ethics approval and consent

The Ethical Review Board in Sweden approved the study (ethics permit number 2019-04746). Written consent to participate was provided by the subject or their legal guardians. The research conformed to the principles of the Helsinki Declaration. Written informed consent was obtained to publish.

### Long-read genome sequencing

The DNA samples were fragmented to 15–20 kb using Megaruptor 3 (Diagenode). PacBio SMRTbell library construction was performed using the SMRTbell Template prep kit 3.0. SMRTbell libraries were size-selected either using AMPure beads or by the gel-based systems pippinHT (Sage Science) or SageElf (Sage Science). The library preparation procedure is described in the protocol “Preparing whole genome and metagenome sequencing libraries using SMRTbell prep kit 3.0” from PacBio. The SMRTbell library sizes and profiles were evaluated using Fragment Analyzer (Agilent Technologies). PacBio sequencing was performed on the PacBio Revio system with 24 h movie time. Each SMRTbell library was sequenced on a 25M SMRT cell.

### Analysis of long-read genome data

The samples were run using tools available in the Nallo pipeline (https://github.com/genomic-medicine-sweden/nallo). Additional analyses were then carried out on top of these results. Briefly, the PacBio HiFi reads were aligned to both GRCh38 and the T2T-CHM13v2.0 reference using minimap2 (version 2.26) (Li 2018) SAMtools (version 1.17) (Danecek et al. 2021). SNVs and INDELs were called with DeepVariant 1.5.0 (Poplin et al. 2018) and phased using WhatsHap 1.7 (Martin et al. 2016). SVs were called using Sniffles 1.1.12 (Sedlazeck et al. 2018), which was run according to previously published parameters (Eisfeldt et al. 2021), and CNVs were called using HiFiCNV (0.1.6b or 0.1.7) (https://github.com/PacificBiosciences/HiFiCNV). Quality metrics were gathered using FastQC 0.11.9 (http://www.bioinformatics.babraham.ac.uk/projects/fastqc/), cramino 0.9.7 (De Coster and Rademakers 2023), and mosdepth 0.3.3 (Pedersen and Quinlan 2018). Phased assemblies were generated using hifiasm (version 0.19.5-r587) (Cheng et al. 2021). The resulting de novo assemblies were aligned to hg38 and T2T-CHM13 reference using minimap2 (version 2.26) (Li 2018), SNVs were called using HTSBOX (version r345) (Danecek et al. 2021) and counted using BCFtools (version 1.17) (Danecek et al. 2021), SVs were called using SVIM-asm using diploid mode for phased assemblies and haploid mode for nonphased assemblies (Heller and Vingron 2021), and quality control was performed using QUAST (version 5.0.2) (Mikheenko et al. 2018).

### Identification and characterization of structural variants

Before SV characterization, the Sniffles SV calls were filtered based on size and allele frequencies. The size filtering was performed using BCFtools (Danecek et al. 2021), excluding all SV calls smaller than 2 kbp. The frequency filtering was performed using SVDB (Eisfeldt et al. 2017). First, a frequency database was constructed using the calls from all 16 individuals sequenced (Table 1). Next, we annotated the remaining calls using that database, and variants present in more than one unrelated individual were removed. Lastly, the remaining SV calls were inspected using IGV (Robinson et al. 2011) and characterized as described previously (Eisfeldt et al. 2021).

## Data access

All lrGS data generated in this study have been submitted to the European Genome-phenome Archive (EGA; https://ega-archive.org) under accession number EGAD50000000676. Code for running the lrGS analysis is available at GitHub (https://github.com/genomic-medicine-sweden/nallo) and as Supplemental Code.

## Supplemental Material

Supplement 1

Supplement 2
