# Supplementary material for: A national long-read sequencing study on chromosomal rearrangements uncovers hidden complexities
Source: Genome Res. 2024 Nov;34(11):1774–84. doi: 10.1101/gr.279510.124 (PMC11610602; doi:10.1101/gr.279510.124)
Supplement: Supplement 1 [file Supplemental_Code.zip › nallo-dev/assets/email_template.html]

genomic-medicine-sweden/nallo Pipeline Report


# genomic-medicine-sweden/nallo ${version}

## Run Name: $runName

<% if (!success){
out << """

#### genomic-medicine-sweden/nallo execution completed unsuccessfully!

The exit status of the task that caused the workflow execution to fail was: `$exitStatus`.

The full error message was:

```
${errorReport}
```

"""
} else {
out << """

genomic-medicine-sweden/nallo execution completed successfully!

"""
}
%>

The workflow was completed at **$dateComplete** (duration: **$duration**)

The command used to launch the workflow was as follows:

```
$commandLine
```

### Pipeline Configuration:

<% out << summary.collect{ k,v -> "|  |  |
| --- | --- |
| $k | ``` $v ``` |
" }.join("\n") %>

genomic-medicine-sweden/nallo

https://github.com/genomic-medicine-sweden/nallo
