# Supplementary figures and images for: A national long-read sequencing study on chromosomal rearrangements uncovers hidden complexities
Source: Genome Res. 2024 Nov;34(11):1774–84. doi: 10.1101/gr.279510.124 (PMC11610602; doi:10.1101/gr.279510.124)

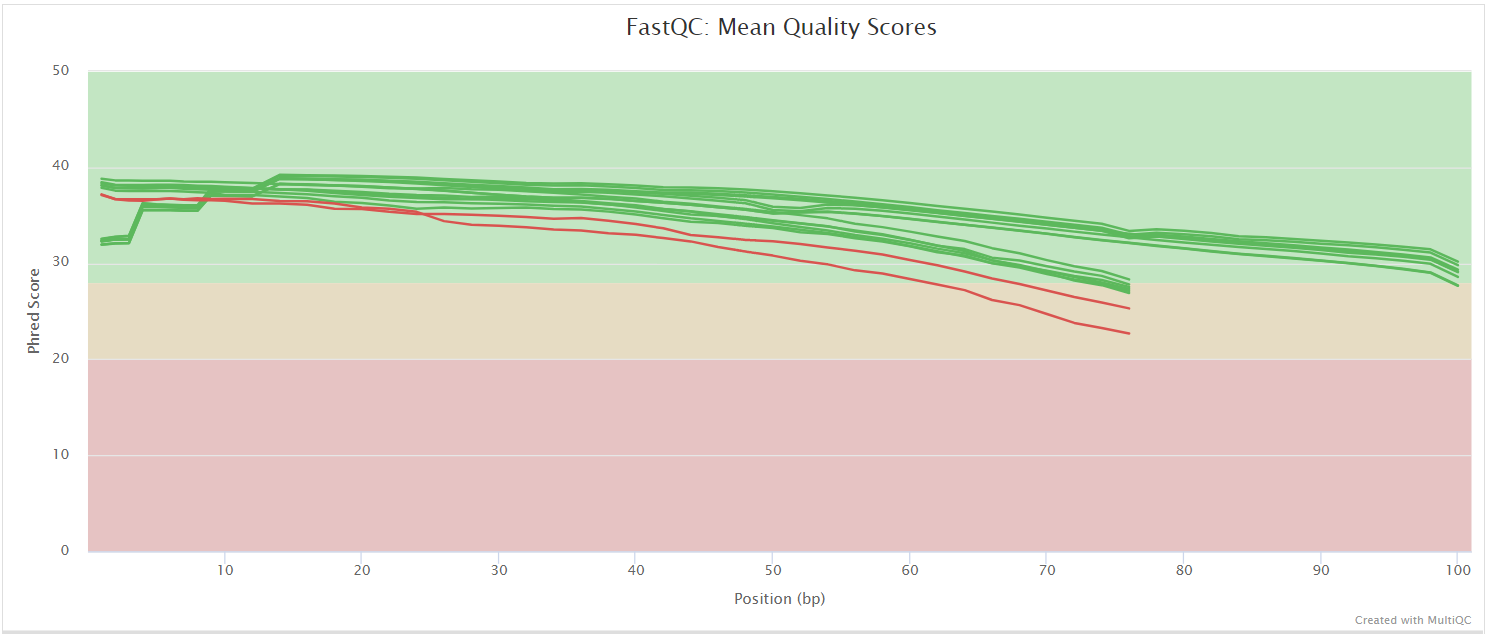

Supplement: Supplement 1 [file Supplemental_Code.zip › nallo-dev/docs/images/mqc_fastqc_quality.png]

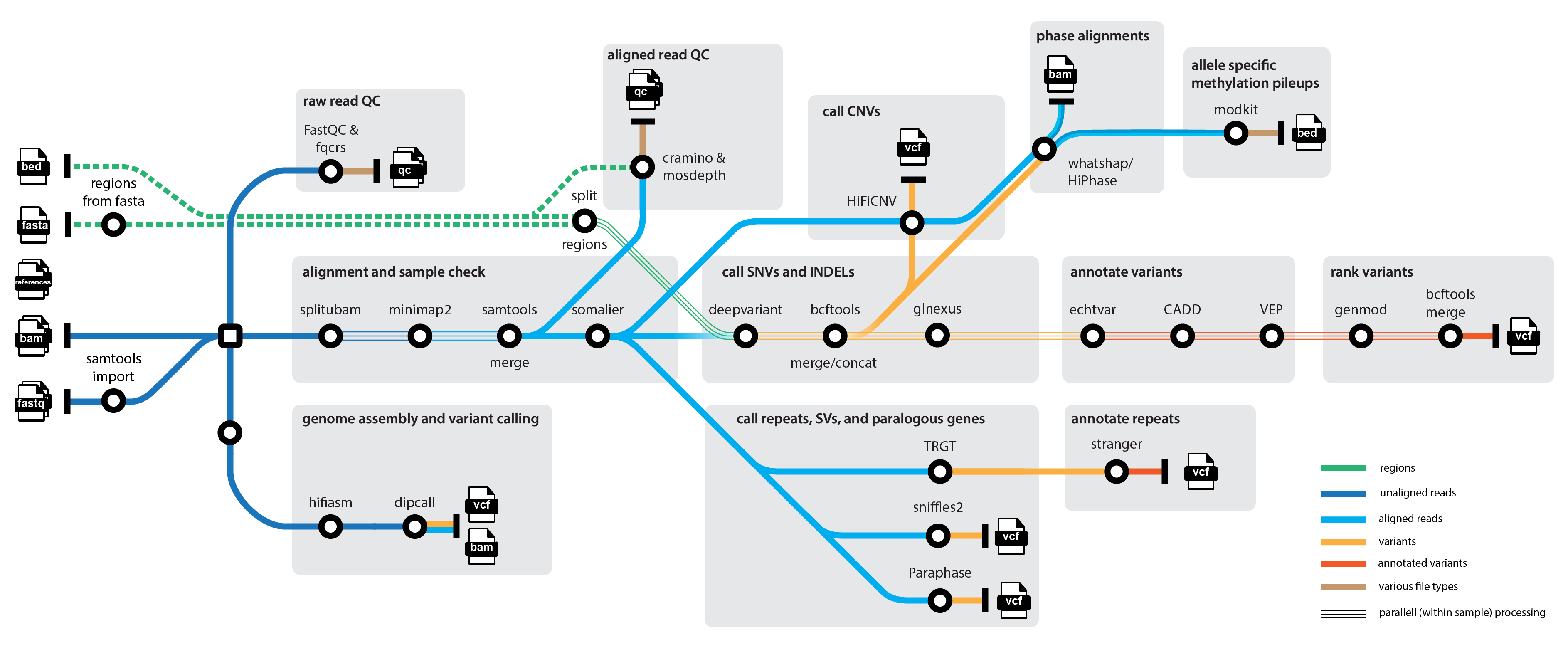

Supplement: Supplement 1 [file Supplemental_Code.zip › nallo-dev/docs/images/nallo_metromap.png]

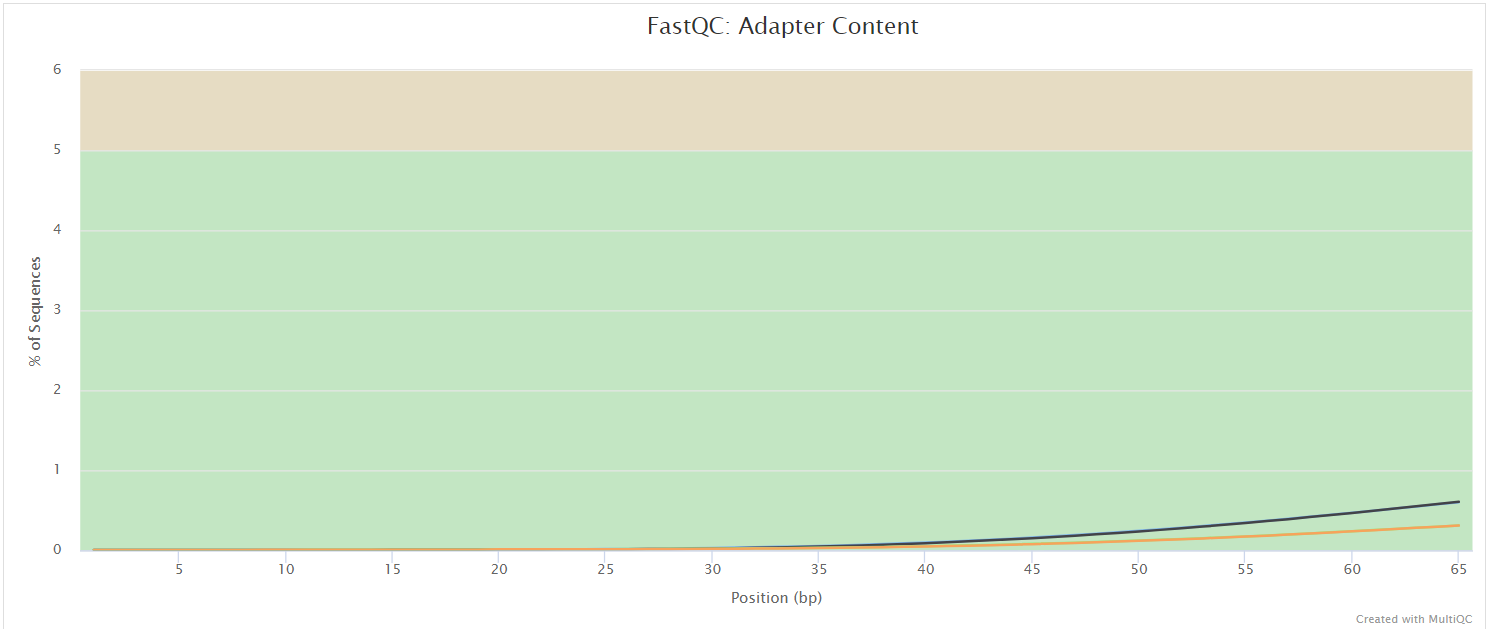

Supplement: Supplement 1 [file Supplemental_Code.zip › nallo-dev/docs/images/mqc_fastqc_adapter.png]

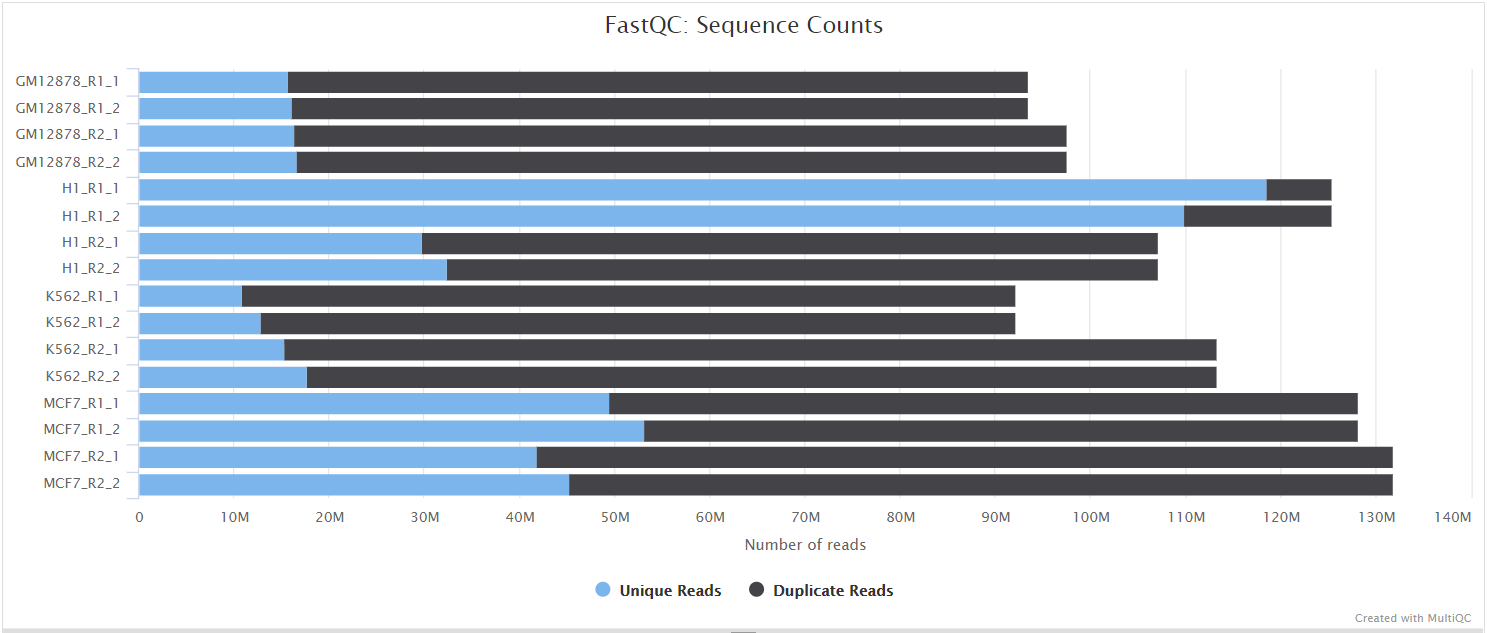

Supplement: Supplement 1 [file Supplemental_Code.zip › nallo-dev/docs/images/mqc_fastqc_counts.png]
