# Supplementary material for: A national long-read sequencing study on chromosomal rearrangements uncovers hidden complexities
Source: Genome Res. 2024 Nov;34(11):1774–84. doi: 10.1101/gr.279510.124 (PMC11610602; doi:10.1101/gr.279510.124)
Supplement: Supplement 2 [file Supplemental_Material.docx]

**Supplemental Tables**

**Supplemental Table S1:** Long-read genome sequencing quality metrics.

| **Sample ID** | **Mean coverage** | **Mean read length (bp)** | **Sequencing Yield (Gb)** |
| --- | --- | --- | --- |
| P1 | 26.5 | 12,307 | 82 |
| P2 | 31.7 | 14,896 | 98 |
| P3 | 27.5 | 12,563 | 85 |
| P4 | 21.2 | 11,233 | 66 |
| P5 | 22.7 | 9,567 | 70 |
| P6 | 19.9 | 8,343 | 62 |
| P7.1 | 31.7 | 14,265 | 98 |
| P7.2 | 25.2 | 12,277 | 78 |
| P7.3 | 25.2 | 12,284 | 78 |
| P8.1 | 32.1 | 15,104 | 99 |
| P8.2 | 31.0 | 17,612 | 96 |
| P9 | 27.5 | 13,285 | 86 |
| P10 | 20.1 | 9,849 | 63 |
| P11 | 27.4 | 15,716 | 90 |
| P12 | 24.9 | 18,810 | 84 |
| P13 | 35.5 | 17,940 | 119 |

Bp, base pairs; Gb, giga bases

**Supplemental Table S2:** De novo assembly quality metrics.

| **Assembly** | **Contigs (n)** | **Total length (bp)** | **N50 (bp)** | **Genome Fraction (%)** | **Small variants (n)** | **SVs (n)** | **Indels** |
| --- | --- | --- | --- | --- | --- | --- | --- |
| P1 hap1 | 952 | 3011730018 | 27656118 | 97.294 | 3573451 | 34337 | 706786 |
| P1 hap2 | 809 | 3020289601 | 27627387 | 97.003 | 3579905 | 11666 | 703549 |
| P1 no hap | 497 | 3055351291 | 39172056 | 97.545 | 3602598 | 33737 | 707649 |
| P2 hap1 | 528 | 3049271102 | 53162323 | 97.431 | 3625230 | 35135 | 713293 |
| P2 hap2 | 607 | 3022576100 | 30089741 | 97.345 | 3639562 | 13701 | 716270 |
| P2 no hap | 373 | 3077093992 | 55656714 | 97.596 | 3665279 | 34006 | 718203 |
| P3 hap1 | 805 | 2968923625 | 38003410 | 95.147 | 3629122 | 35288 | 714993 |
| P3 hap2 | 683 | 2955832984 | 26901484 | 94.768 | 3608950 | 14089 | 709099 |
| P3 no hap | 470 | 3070113002 | 57555714 | 97.867 | 3727047 | 32916 | 732983 |
| P4 hap1 | 2167 | 3009507143 | 10032076 | 96.786 | 3543724 | 33,371 | 723511 |
| P4 hap2 | 1870 | 2994360817 | 10443380 | 96.707 | 3600158 | 11455 | 729278 |
| P4 no hap | 990 | 3061337924 | 31471136 | 97.581 | 3649743 | 33158 | 734887 |
| P5 hap1 | 1410 | 3021926484 | 16791079 | 97.001 | 3564965 | 33505 | 710273 |
| P5 hap2 | 1442 | 3002452954 | 10513426 | 96.988 | 3559703 | 11743 | 710897 |
| P5 no hap | 780 | 3050115512 | 32705197 | 97.460 | 3621814 | 33780 | 717110 |
| P6 hap1 | 2891 | 2990264780 | 5665656 | 96.073 | 3589314 | 33140 | 734226 |
| P6 hap2 | 2786 | 2894053352 | 5660041 | 93.124 | 3516636 | 10247 | 718105 |
| P6 no hap | 1092 | 3059225159 | 20150227 | 97.822 | 3667936 | 32845 | 746927 |
| P7.1 hap1 | 543 | 3038562420 | 44240849 | 97.376 | 3620569 | 35664 | 708884 |
| P7.1 hap2 | 516 | 3037726858 | 49152777 | 97.415 | 3627346 | 14683 | 709222 |
| P7.1 no hap | 387 | 3070887463 | 70906275 | 97.538 | 3650945 | 34901 | 710859 |
| P7.2 hap1 | 1098 | 3004917728 | 26806592 | 96.139 | 3581699 | 34708 | 714973 |
| P7.2 hap2 | 909 | 2994455258 | 23607483 | 95.712 | 3581148 | 14310 | 715250 |
| P7.2 no hap | 579 | 3083744197 | 43485889 | 98.143 | 3649458 | 34144 | 727696 |
| P7.3 hap1 | 919 | 3007113736 | 29658224 | 97.315 | 3602776 | 35187 | 716780 |
| P7.3 hap2 | 785 | 3029744114 | 24927346 | 97.265 | 3628930 | 13774 | 716232 |
| P7.3 no hap | 514 | 3055007195 | 53717007 | 97.582 | 3660593 | 34144 | 720613 |
| P8.1 hap1 | 541 | 3024919811 | 56515313 | 97.460 | 3627237 | 35517 | 708068 |
| P8.1 hap2 | 571 | 3025639302 | 38174944 | 97.279 | 3594413 | 15832 | 706405 |
| P8.1 no hap | 367 | 3065271514 | 69545151 | 97.632 | 3643325 | 36161 | 709464 |
| P8.2 hap1 | 628 | 3027302868 | 62105149 | 97.384 | 3621918 |  | 706580 |
| P8.2 hap2 | 526 | 3015816193 | 49998385 | 97.365 | 2469965 | 4138 | 491224 |
| P8.2 no hap | 415 | 3058931416 | 73038069 | 97.708 | 3667377 | 33791 | 711103 |
| P9 hap1 | 655 | 3034915555 | 54793259 | 97.408 | 3600299 | 33117 | 711583 |
| P9 hap2 | 706 | 3025770706 | 28512459 | 97.274 | 3570230 | 12307 | 2861175 |
| P9 no hap | 554 | 3066133149 | 53797994 | 97.617 | 3604444 | 33028 | 712078 |
| P10 hap1 | 2742 | 3014608061 | 8875707 | 96.653 | 3548895 | 35094 | 722959 |
| P10 hap2 | 2623 | 2886636947 | 7067025 | 92.984 | 3473909 | 10671 | 704156 |
| P10 no hap | 1301 | 3062985831 | 27709462 | 97.976 | 3617165 | 29860 | 732080 |
| P11 hap1 | 940 | 3033114741 | 40056718 | 96.571 | 3621609 | 34036 | 711084 |
| P11 hap2 | 884 | 2978655919 | 31312892 | 95.787 | 3525896 |  | 699382 |
| P11 no hap | 597 | 3051400356 | 62335257 | 97.275 | 3647060 | 33749 | 714031 |
| P12 hap1 | 1300 | 3036918378 | 23705991 | 96.653 | 3610216 | 34670 | 750021 |
| P12 hap2 | 1047 | 2916967371 | 21936164 | 93.432 | 3550325 |  | 730708 |
| P12 no hap | 746 | 3088070636 | 40766497 | 97.750 | 3699510 | 32626 | 759519 |
| P13 hap1 | 305 | 3021367031 | 58169784 | 96.398 | 3602439 | 34989 | 707153 |
| P13 hap2 | 278 | 2936874730 | 60498075 | 93.783 | 3563910 |  | 694026 |
| P13 no hap | 276 | 3101975158 | 85852130 | 97.896 | 3677600 | 36840 | 716890 |

**Supplemental Table S3**: MultiQC.

Cov., Coverage; Vars, Variations total; SNP, Variation single nucleotide polymorphisms (SNPs); Indel, Variation insertions/deletions; Ts/Tv, variant SNP transition/transversion ratio; % Dups, Duplicate reads; M Seqs, Total sequences (millions); Avg, Average

**Supplemental Table S4:** Number of SNV calls compared to GnomAD.

| CaseID | Total | In GnomAD | % | Not in GnomAD | % |
| --- | --- | --- | --- | --- | --- |
| P1 | 4734962 | 4542063 | 96% | 192899 | 4% |
| P2 | 4812148 | 4602457 | 96% | 209691 | 4% |
| P3 | 4760088 | 4556324 | 96% | 203764 | 4% |
| P4 | 4673347 | 4495067 | 96% | 178280 | 4% |
| P5 | 4723037 | 4538478 | 96% | 184559 | 4% |
| P6 | 4710397 | 4535321 | 96% | 175076 | 4% |
| P7.1 | 4826412 | 4614965 | 96% | 211447 | 4% |
| P7.2 | 4717344 | 4525412 | 96% | 191932 | 4% |
| P7.3 | 4773762 | 4574527 | 96% | 199235 | 4% |
| P8.1 | 4797782 | 4592086 | 96% | 205696 | 4% |
| P8.2 | 4774075 | 4562894 | 96% | 211181 | 4% |
| P9 | 4651202 | 4477303 | 96% | 173899 | 4% |
| P10 | 4726621 | 4522039 | 96% | 204582 | 4% |
| P11 | 4764356 | 4558042 | 96% | 206314 | 4% |
| P12 | 4666255 | 4459085 | 96% | 207170 | 4% |
| P13 | 4849424 | 4617296 | 95% | 232128 | 5% |

SNV, single nucleotide variants; INDELS, insertions/deletions; GnomAD, genome aggregation database

**Supplemental Table S5:** De novo assembly variant calling.

| **Sample ID** | **ChrA** | **posA** | **chrB** | **posB** | **Found?** |
| --- | --- | --- | --- | --- | --- |
| P1 | 4 | 181414917 | 9 | 13043905 | Yes, in both haps |
|  | 16 | 28454138 | 16 | 29 482 543 | Yes, hap 2 |
| P2 | - | - | - | - | - |
| P3 | 22 | 50618055 | 22 | 50624868 | Yes, hap1 |
|  | 22 | 50624361 | 22 | 50626277 | Yes, hap 1 |
| P4 | 2 | 5549092 | 2 | 5858296 | Yes, hap2 |
|  | 2 | 5620671 | 2 | 7241289 | No |
|  | 2 | 6246303 | 2 | 7734100 | Yes, hap2 (called as del) |
|  | 2 | 6248808 | 2 | 7246507 | Yes, both haps |
|  | 2 | 132954122 | 2 | 137703180 | Yes, hap2 |
|  | 2 | 133434126 | 2 | 141163928 | Yes, hap2 |
| P5 | 3 | 54518907 | 3 | 63614153 | Yes, hap2 |
|  | 3 | 59407900 | 3 | 135767535 | Yes, hap2 |
|  | 3 | 63614153 | 3 | 145600155 | Yes, hap1 |
|  | 3 | 80981660 | 3 | 135891958 | Yes, hap2 |
|  | 3 | 80981660 | 3 | 148110490 | Yes, hap2 |
| P6 | 9 | 97596764 | X | 153779639 | Not found, but calls a deletion on X between the two breakpoints shown here |
|  | 9 | 97598236 | X | 153724706 |  |
| P7.1 | 1 | 19783172 | 10 | 95395335 | Yes, hap1 |
|  | 1 | 19783105 | 10 | 95395328 | Yes, hap2 |
|  | 2 | 189694535 | 2 | 202576085 | Yes, hap2 |
|  | 2 | 189694533 | 2 | 202576083 | Yes, hap2 |
| P7.2 | - | - | - | - | - |
| P7.3 | 1 | 19783172 | 10 | 95395335 | Yes, hap2 |
|  | 1 | 19783105 | 10 | 95395328 | Yes, hap2 |
|  | 2 | 189694535 | 2 | 202576085 | Yes, hap1 |
|  | 2 | 189694533 | 2 | 202576083 | Yes, hap1 |
| P8.1 | X | 9420014 | X | 154113037 | Yes, hap2 |
|  | X | 9768909 | X | 154208530 | No |
| P8.2 | X | 9420014 | X | 154113037 | Yes, non-phased |
|  | X | 9768909 | X | 154208530 | Yes, BPJ 1: 9768884  BPJ 2: 154208531 |
| P9 | 9 | 75862011* | X | 3044233 | Yes, hap 2. BPJ 1: 75862276 |
|  | 9 | 75862011* | X | 3044228 | Yes, hap2. BPJ 1: 75862011 |
| P10 | - | - | - | - | - |
| P11 | X | 101431832 | X | 55349282 | Yes, hap2 |
| P12 | 4 | 80390384 | 6 | 20052376 | Yes, hap2 |
|  | 1 | 58205788 | 4 | 106505089 | Yes, in both haps |
|  | 6 | 20052374 | 4 | 94218568 | Yes, in both haps |
|  | 4 | 106505092 | 1 | 58205785 | Yes, hap1 |
|  | 6 | 48002694 | 6 | 49160076 | Yes, hap2 |
| P13 | - | - | - | - | - |

**Supplemental Table S6:** Number of SV calls, filtered and unfiltered.

| Case  ID | All Calls | | | | | Unique Calls | | | | | Unique Calls affecting exons in genelist | | | |
| --- | --- | --- | --- | --- | --- | --- | --- | --- | --- | --- | --- | --- | --- | --- |
|  | Intrachr | | | | Interchr | Intrachr | | | | Interchr | Intrachr | | | |
|  | 100bp – 1kbp | 1kbp – 10kbp | 10kbp – 100kbp | > 100kbp | 100bp – inf | 100bp – 1kbp | 1kbp – 10kbp | 10kbp – 100kbp | > 100 kbp | 100bp – inf | 100bp – 1kbp | 1kbp – 10kbp | 10kbp – 100kbp | > 100 kbp |
| P1 | 12844 | 2983 | 258 | 270 | 628 | 977 | 216 | 11 | 8 | 61 | 0 | 0 | 0 | 4 |
| P2 | 13254 | 3305 | 331 | 342 | 716 | 1050 | 276 | 17 | 31 | 63 | 1 | 1 | 0 | 4 |
| P3 | 13075 | 3134 | 328 | 303 | 673 | 1391 | 313 | 23 | 15 | 61 | 1 | 3 | 0 | 4 |
| P4 | 12233 | 2676 | 209 | 226 | 539 | 903 | 188 | 18 | 17 | 49 | 0 | 0 | 0 | 5 |
| P5 | 12432 | 2746 | 244 | 255 | 543 | 921 | 201 | 14 | 23 | 35 | 0 | 0 | 0 | 14 |
| P6 | 13322 | 3257 | 324 | 378 | 842 | 755 | 173 | 20 | 21 | 68 | 0 | 0 | 0 | 10 |
| P7.2 | 13113 | 3005 | 304 | 288 | 663 | 1081 | 252 | 12 | 7 | 49 | 0 | 0 | 0 | 1 |
| P7.3 | 12936 | 2998 | 292 | 297 | 688 | 1025 | 250 | 24 | 24 | 71 | 0 | 0 | 0 | 10 |
| P8.1 | 13185 | 3090 | 319 | 346 | 779 | 1004 | 223 | 8 | 18 | 101 | 0 | 0 | 0 | 9 |
| P9 | 12245 | 2625 | 226 | 234 | 539 | 936 | 183 | 14 | 16 | 46 | 2 | 0 | 0 | 10 |
| P10 | 12899 | 3107 | 298 | 325 | 620 | 984 | 254 | 14 | 17 | 59 | 1 | 0 | 0 | 6 |
| P11 | 12819 | 3041 | 256 | 266 | 625 | 965 | 248 | 14 | 18 | 64 | 1 | 0 | 0 | 6 |
| P12 | 12583 | 3029 | 275 | 261 | 534 | 1130 | 302 | 17 | 16 | 39 | 3 | 0 | 1 | 3 |
| P13 | 13664 | 3478 | 425 | 408 | 831 | 1358 | 342 | 31 | 25 | 96 | 0 | 0 | 1 | 11 |

Intrachr, Intrachromosomal; Interchr, Interchromosomal; Inf, infinity; Bp, base pairs; Kbp, kilo-base pairs

**Supplemental Table S7:** DNA extraction methods.

| CaseID | FemtoPulse | | | | Extraction method | Manufacturer |
| --- | --- | --- | --- | --- | --- | --- |
|  | 300 bp - 10 kbp | 10 kbp - 25 kbp | 25 kbp - 300 kbp |  | |  |
| P1 | 8,10% | 12,80% | 77,30% | QiaSymphony (bead-based) | | QIAGEN |
| P2 | 3,80% | 8,40% | 84,90% | Qiacube (Qiagen blood mini kit) | | QIAGEN |
| P3 | 6,00% | 20,20% | 73,60% | Qiagen blood mini kit (Manual extraction) | | QIAGEN |
| P4 | 10,80% | 14,60% | 74,00% | QiaSymphony (bead-based) | | QIAGEN |
| P5 | 13,90% | 30,00% | 52,80% | QIAamp DNA Blood Mini Kit (Manual extraction) | | QIAGEN |
| P6 | 12,00% | 31,50% | 55,80% | QIAamp DNA Blood Mini Kit (Manual extraction) | | QIAGEN |
| P7.1 | 1,80% | 3,00% | 93,20% | SP Blood & cell culture DNA isolation kit (Manual extraction) | | Bionano genomics |
| P7.2 | 1,90% | 2,30% | 94,20% | SP Blood & cell culture DNA isolation kit (Manual extraction) | | Bionano genomics |
| P7.3 | 16,30% | 7,50% | 74,10% | SP Blood & cell culture DNA isolation kit (Manual extraction) | | Bionano genomics |
| P8.1 | 3,70% | 9,80% | 85,60% | Nanobind CBB kit (Manual extraction) | | Pacific biosciences |
| P8.2 | 4,30% | 10,60% | 83,80% | Nanobind CBB kit (Manual extraction) | | QIAGEN |
| P9 | 3,80% | 13,00% | 79,80% | Qiagen EZ1 | | QIAGEN |
| P10 | 11,40% | 20,60% | 52,00% | QiaSymphony (bead-based) | | QIAGEN |
| P11 | 23,50% | 45,50% | 31,20% | Chemagic 360 (bead-based) | | Perkin Elmer |
| P12 | 17,40% | 16,80% | 65,70% | Chemagic 360 (bead-based) | | Perkin Elmer |
| P13 | 27,70% | 40,60% | 23,30% | QiaSymphony (bead-based) | | QIAGEN |

Bp, base pairs; kbp, kilo base pairs

**Supplemental Figures**

**Supplemental Fig S1: Read coverage variation across chromosome 15.** Average read coverage per 1 MB segments of chromosome 15 for sample P2.


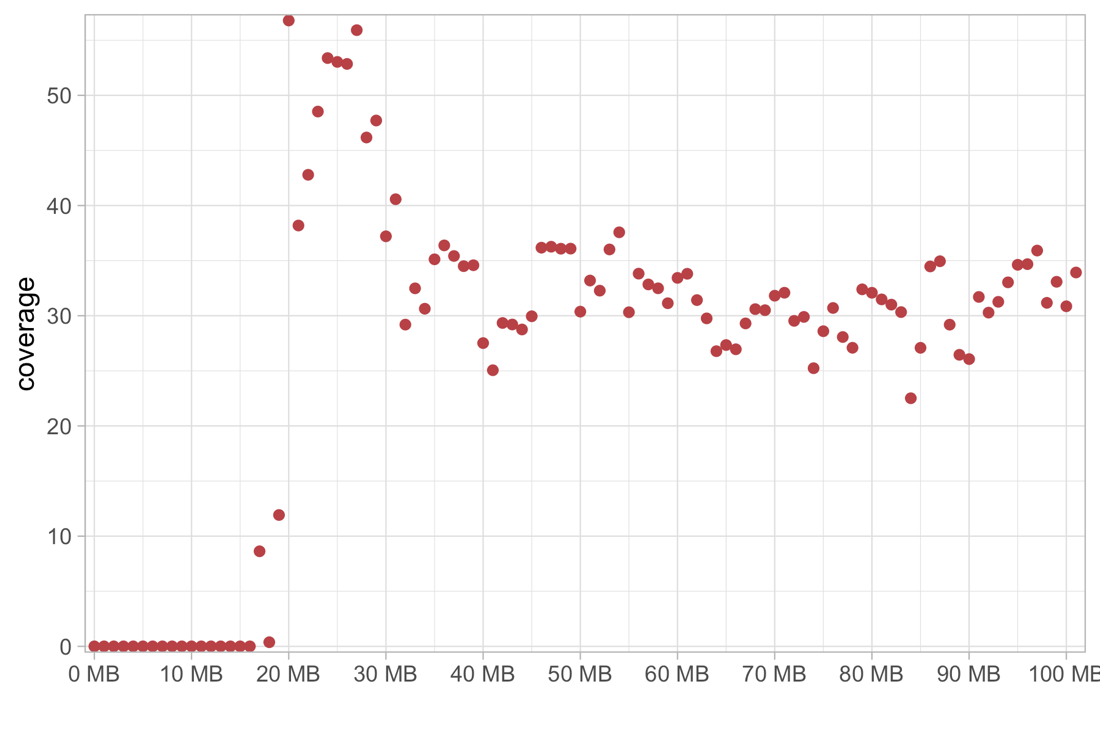


**Supplemental Fig S2: Subway plots of characterized rearrangements.** Schematic illustrations of the resolved rearrangements in P3, P6, P4, P11 and P8. Red and pink segments are deleted in the patients and arrows show the inverted segments. Der: derivative chromosome.


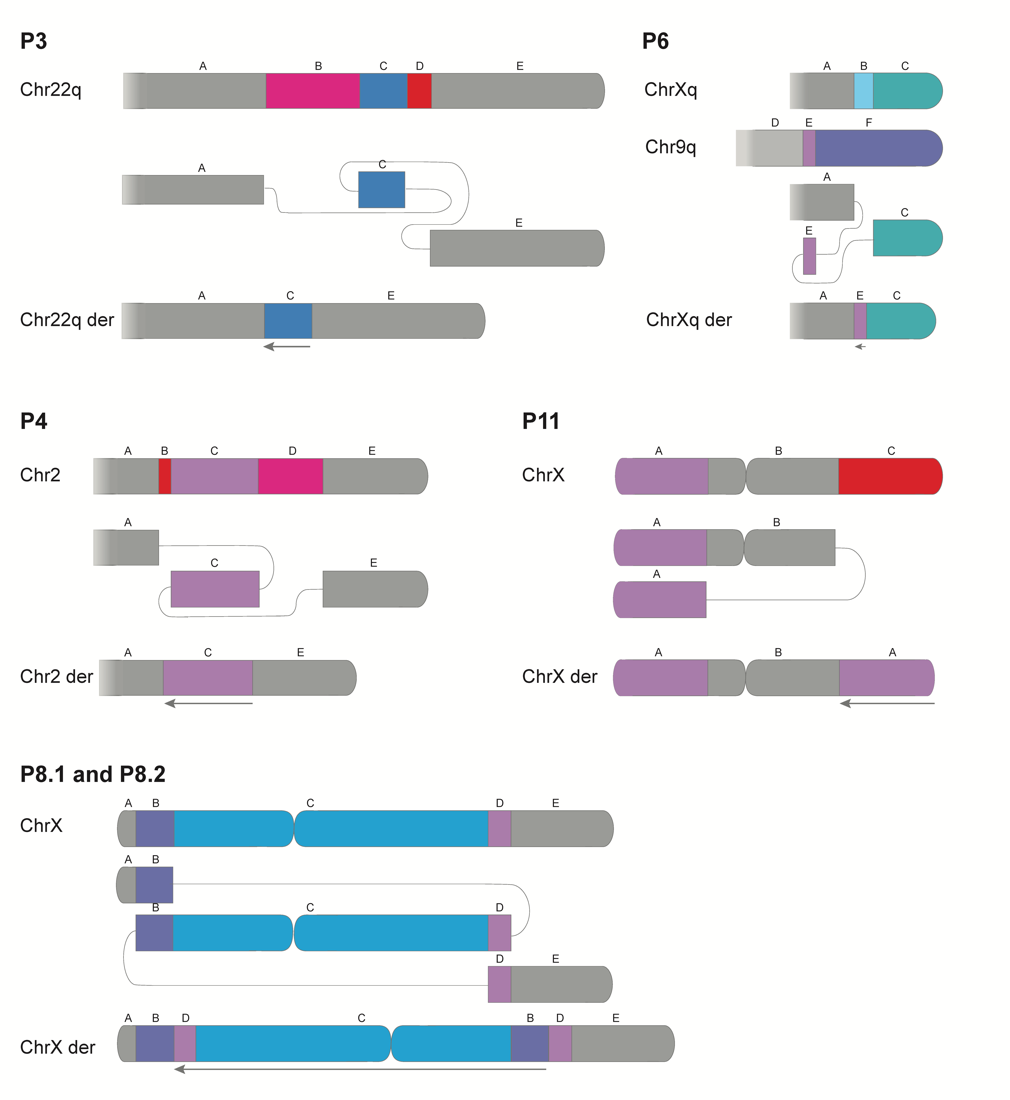


**SUPPLEMENTARY REFRENCES**

1. Danecek P, Bonfield JK, Liddle J, Marshall J, Ohan V, Pollard MO, et al. Twelve years of SAMtools and BCFtools. Gigascience. 2021;10(2).

2. Li H. Minimap2: pairwise alignment for nucleotide sequences. Bioinformatics. 2018;34(18):3094-100.

3. Poplin R, Chang PC, Alexander D, Schwartz S, Colthurst T, Ku A, et al. A universal SNP and small-indel variant caller using deep neural networks. Nat Biotechnol. 2018;36(10):983-7.

4. Martin M, Patterson M, Garg S, Fischer SO, Pisanti N, Klau GW, et al. WhatsHap: fast and accurate read-based phasing. bioRxiv. 2016:085050.

5. De Coster W, Rademakers R. NanoPack2: population-scale evaluation of long-read sequencing data. Bioinformatics. 2023;39(5).

6. Pedersen BS, Quinlan AR. Mosdepth: quick coverage calculation for genomes and exomes. Bioinformatics. 2018;34(5):867-8.

7. Cheng H, Concepcion GT, Feng X, Zhang H, Li H. Haplotype-resolved de novo assembly using phased assembly graphs with hifiasm. Nat Methods. 2021;18(2):170-5.
